# Critical care response to a hospital outbreak of the 2019-nCoV infection in Shenzhen, China

**DOI:** 10.1186/s13054-020-2786-x

**Published:** 2020-02-19

**Authors:** Yong Liu, Jinxiu Li, Yongwen Feng

**Affiliations:** 1 Department of Critical Care Medicine, Shenzhen Hospital, Southern Medical University, Shenzhen, Guangdong China; 2 Expert panel of Shenzhen 2019-nCoV pneumonia, Shenzhen, Guangdong China; 3 Department of Critical Care Medicine, Shenzhen Third People’s Hospital, Shenzhen, Guangdong China; 4 Department of Critical Care Medicine, Shenzhen Second People’s hospital, No 3002, Sungang Road, Futian District, Shenzhen, 518028 Guangdong China

Beginning at early December 2019, there is an outbreak of a novel 2019-nCoV in Wuhan, China. Then, Public Health Emergency of International Concern (PHEIC) was declared on 30 January 2020, by the World Health Organization (WHO) Emergency Committee. The 2019-nCoV pandemic could lead to an influx of critically ill into the already strained hospital systems in mainland China. Dealing with the pandemic requires a robust hospital- and city-wide command and control structure that could make quick and informed decisions among Chinese hospitals. We recommend that a proper plan can enable the local government, healthcare systems, hospitals, and healthcare workers to better cope with such public eventuality. The response should be flexible and adjustable according to the size of the population impacted.

## The main challenge

The main challenge may include (1) early identification of outbreak, (2) rapid expansion of patients, (3) high risk of nosocomial transmission, (4) unpredictability of size impacted, and (5) lack of backup resource. These challenges have caused severe shortage of healthcare workers, medical materials, and beds with isolation. The Spring Festival holiday has greatly aggravated the shortage of human resources and heavy traffic flow due to the vacation of healthy workers and factory workers, which further magnified the risk of transmission. The key point is to discriminate the infectious disease outbreak from regular clustering cases of flu-like diseases at early stage. There is a trade-off between false alarm causing population panic and delayed identification leading to social crisis.

## Clinical features

Early identification of 2019-nCoV infection presents a major challenge for the frontline clinicians. Its clinical symptoms largely overlap with those of common acute respiratory illnesses, including fever (98%), cough (76%), and diarrhea (3%), often more severe in older adults with pre-existing chronic comorbidities [1]. Usually, the laboratory abnormalities include lymphocytopenia and hypoxemia [1]. The initial chest radiographs may vary from minimal abnormality to bilateral ground-glass opacity or subsegmental areas of consolidation [1]. In addition, asymptomatic cases and lack of diagnosis kits result in delayed or even missed diagnosis inevitable and makes many other patients, visitors, and healthcare workers exposed to the 2019-nCoV infection.

## Critical care response at the hospital and city level

### City level

Critical care response to the outbreak of coronavirus should happen not only at the level of hospital, but also at the level of the city which is dominated by the government. At the early stage, the size of the patients’ population is not beyond the capability of local infectious diseases hospital (IDH). The general hospital is responsible for fever triage, identifying suspected cases, and transferring to the local IDH. Such a plan is mandatory for every hospital. Shenzhen city has established a preexisting Infectious Disease Epidemic Plan (IDEP), which has facilitated managing and containing local outbreak of the 2019-nCoV. In case the patient load exceeds the hospital capability of the IDH, new IDHs should be considered either by building a temporary new IDH or reconstructing an existing hospital. Wuhan, the epicenter of the outbreak, is racing against time to build two specialized hospitals for nCoV patients, namely Huoshenshan and Leishenshan hospital, whereas a different strategy has been undertaken in Shenzhen city by reconstructing an existing hospital to become an IDH with capability of 800 beds.

### Hospital level

2019-nCoV patients should be admitted to single-bedded, negative pressure rooms in isolated units with intensive care and monitoring [2]. Clinical engineering should have plans to reconstruct standard rooms [2]. Retrofitting the rooms with externally exhausted HEPA filters may be an expedient solution. Also, the general hospital may consider procedures such as suspending elective surgeries, canceling ambulatory clinics and outpatient diagnostic procedures, transferring patients to other institutions, and restricting hospital visitors [2]. More importantly, because the hospitals’ ability to respond to the outbreak largely depends on their available ICU beds, the plan to increase ICU bed capacity needs to be determined.

## Key points for preventing transmission of infectious agents in healthcare settings

Caring for 2019-nCoV patients represents a substantial exposure risk for ICU staff because of the following reasons: highly contagious with multiple transmission route, high exposure dose, long daily contact hours, and ICU stay. The basic reproductive number was estimated to be 2.2 (95% CI, 1.4 to 3.9) [3], or as high as between 3.6 and 4.0 [4]. The 2019-nCoV is proved to be transmitted by respiratory droplets, contact, and fecal-oral, even transmission through the eye is possible [5, 6]. The higher viral load and aerosol-generating procedures, such as non-invasive ventilation, magnify the exposure and transmission risk [2, 7, 8]. Moreover, virus shedding can be prolonged and last for > 3 weeks according to some literature and our unpublished data [2]. Healthcare providers and those in contact with infected patients should utilize contact, droplet, and airborne precautions with N95 respirator. Strict infection prevention and control practices have been implemented and audited in our units following the infection prevention and control plan published by China’s National Health Committee (CNHC). In addition, well-equipped fever clinic as triage station with trained staff knowing 2019-nCoV case definitions is established. For suspected 2019-nCoV infection, several key points are crucial procedures: recording a detailed history, standardizing pneumonia workup, obtaining lower respiratory tract specimens [2, 8], and implementing droplet isolation to break the transmission chain in the healthcare setting [2].

## Psychosocial stress

The risk of 2019-nCoV exposure may cause significant psychosocial stress on healthcare workers [2]. The death of a retired ENT physician from a 2019-nCoV infection has added to fears in January 2020. Psychotherapists have also been invited to join medical teams to evaluate and deal with potential stress and depression for the safety of the healthcare workers.

## Critical management

2019-nCoV management was largely supportive, including intubation, early prone positioning, neuromuscular blockade, and extracorporeal membrane oxygenation (ECMO) according to the recommendations updated by CNHC. Low-dose systematic corticosteroids, lopinavir/ritonavir, and atomization inhalation of interferon were encouraged. These critical managements have worked well so far, as our 2019-nCoV patients had zero mortality. On the contrary, the previously reported mortality of 2019-nCoV patients in Wuhan ranged from 11 to 15% [1, 9].

## Data Availability

Not applicable.
